# 

**Published:** 1897-10-09

**Authors:** 


**Notices of Births, Deaths, and Marriages** are inserted in this column at a charge of 2s. 6d. for an announcement not exceeding four lines.

### NOTICE TO CORRESPONDENTS.

All MS., Letters, Books for Review, and other matters intended for the Editor should be addressed The Editor, "The Hospital," The Hospital Building, 28 & 29, Southampton Street, Strand, W.C.

The Editor cannot undertake to return rejected MS., even when accompanied by stamped directed envelope.

**Notice to Advertisers and Subscribers.**

All Advertisements, Orders for Copies of The Hospital, and Business Communications should be addressed to **THE MANAGER, (Not to The Editor),** The Hospital, The Hospital Building, 28 & 29, Southampton Street, Strand, London, W.C.

The Cost of Subscription, post free, is as follows:—Per week, 2½d.; per quarter, 2s. 8d.; per half-year, 5s. 3d.; per annum, 10s. 6d. Foreign:—Per annum, 15s. 2d.; payable, in all cases, in advance.

**NOTICE.—Vol. XXII. of "The Hospital" (April, 1897, to September, 1897), in handsome cloth binding, is now on sale at the Office of this Journal, price 7s. 6d. Cases for binding the Numbers contained in this Volume, 1s. 6d. Index to Vol. XXII., 2½d., post free. The Monthly Part for October is now ready, Price 6d., by post 8d.**

### BOOKS RECEIVED.

J. and A. Churchill.

"Elements of Human Physiology." By Ernest H. Starling, M.D., F.R.C.P.

Baillière, Tindall, and Cox.

"The Oxygen Treatment." By George Stoker, M.R.C.P.I.

"The Röntgen Rays in Medical Work." By David Walsh, M.D., with an introductory section upon "Electrical Apparatus and Methods" by J. E. Greenhill.

Isbister and Co.

"Perpetua." By S. Baring-Gould, M.A.

Adam and Charles Black.

"The Nurses' Handbook of Cookery." By E. M. Worsnop, assisted by M. C. Blair.

Kegan Paul, Trench, Trubner, and Co.

"Memory and its Cultivation." By F. W. Edridge-Green, M.D., F.R.C.P.

H. K. Lewis.

"Ringworm and Alopecia Areata." By H. Aldersmith, M.B.Lond., F.R.C.S. (Fourth edition.)

Smith, Elder, and Co.

"Spinal Caries." By Noble Smith, F.R.C.S.Ed. (Second edition.)

Longmans, Green, and Co.

"The Dwelling House." By George Vivian Poore, M.D., F.R.C.P.

The Roxburghe Press.

"Young Men's Guide." By Dr. Gangadin.

Cassell and Co.

"Clinical Methods." By Robert Hutchinson, M.D., M.R.C.P., and Harry Rainey, M.A., F.R.C.P.Ed.

The New York Medical Journal.

"The Serum Diagnosis of Enteric Fever by the Dried Blood Method." By J. C. Da Costa, jun., M.D. A reprint.

**Periodicals and Pamphlets.**—Golden Penny, Woman's Signal, Trained Nurse, Medical Record, Local Government Journal, Guy's Hospital Gazette, Literary Digest, Medical Pioneer, The Sun, New Age, Westminster Review, Industries and Iron, The Scalpel, Sunday at Home, The Leisure Hour, Boy's Own Paper, Electrical Review.

### Scraps and Gleanings.

A laundry has been added to the North Lonsdale Hospital.

Mr. E. E. Crisp has been appointed secretary of the Coventry and Warwickshire Hospital.

During 1896 54 men, 81 women, and 25 children were admitted to the Oriolet Cottage Hospital at Loughton.

Two hundred and ninety in, and 1,760 out-patients, were treated at the North Lonsdale Hospital in 1896-7.

A new military hospital is to be erected on the west end of the Castle Rock at Edinburgh at a cost of about £18,000.

The Ely Dispensary Committee report a serious diminution in the income of the institution. Steps are being taken to appeal to the city.

The amounts collected by the Weymouth Hospital Sunday Committee in 1896 and 1897 were surprisingly equal, being £1,118 5s. 7d. and £1,118 13s. 2d. respectively.

The Inspector-General of Gaols in Burma, in his report on the Rangoon Asylum, says that insanity is far more generally prevalent in Burma than elsewhere in India.

Three hundred and fifty-one in-patients were admitted to, and 557 out-patients were treated at, the Royal Cornwall Infirmary in 1896-7. The latter show a satisfactory decrease of 70.

The attempt to induce the cycle firms of Coventry to follow the example of their employés by becoming annual subscribers to the hospital does not appear to be as successful as it deserves.

Altogether 3,950 convalescents were admitted to the West of Scotland Convalescent Homes at Dunoon during the year just ended. These comprised 1,828 men, 1,782 women, 166 boys, and 174 girls.

We are informed that the Victoria Date Co., Ltd., of 112, Belvedere Road, Lambeth, S.E., has just appointed Messrs. Samuel Hannah and Co., of Rutherglen Road, Glasgow, its sole wholesale agents for Scotland for their well-known Date Vinegar, Pickles, &c.

The City of Winchester contributes, roughly speaking, about 25 per cent. of the ordinary income of the Royal Hants County Hospital, whilst it receives quite 75 per cent. of the ordinary expenditure and provides about one-half of the patients treated.

### The Student of Medicine.

### APPOINTMENTS.

B. Addenbrooke, M.B., B.S.Durh., appointed Medical Officer to the Kidderminster Rural District Council. G. Ashton, M.B., appointed an Assistant Medical Officer to the Chorlton Union Workhouse. F. D. Bennett, M.R.C.S., L.R.C.P., L.S.A., appointed Junior Medical Officer to the Army and Navy Co-operative Societies. W. H. Boger, L.R.C.P.Edin., M.R.C.S., appointed Medical Officer to the Lanteglos District of the Liskeard Union. J. S. F. Clark, M.R.C.S.Eng., L.R.C.P.Edin., appointed Medical Officer for the Moretonhampstead District of the Newton Abbot Union. J. E. H. Davies, M.R.C.S.Eng., L.R.C.P.Lond., L.S.A.Lond., appointed Honorary Surgeon to Wrexham Infirmary; Medical Officer of Health for the Northern Division of the Wrexham Rural District Council; Medical Officer to the Wrexham Fever Hospital; and Certifying Factory Surgeon for the Wrexham District. N. Elrington, L.S.A., M.R.C.S., appointed Medical Officer for the Wethersfield District of the Braintree Union. H. L. Ewens, M.D., B.S.Univ.Durh., M.R.C.S.Eng., L.R.C.P.Lond., appointed Medical Officer and Public Vaccinator to the St. Peter's District of the Maldon Union. H. H. Fisher, M.D.Lond., M.R.C.S.Eng., appointed Medical Officer for the No. 2 District of the Milton Union. R. W. Fisher, M.R.C.S.Eng., L.R.C.P.Lond., appointed Medical Officer to the Workhouse and the First District of the Westbury-on-Severn Union. E. J. Fox., B.Sc.Lond., M.R.C.S.Eng., L.R.C.P.Lond., appointed House Surgeon to the Manchester Southern Hospital for Diseases of Women. T. Goldie-Scot, M.B.Edin., appointed Assistant Medical Officer to the Warneford Asylum, Oxford. H. T. Hamilton, L.S.A.Lond., appointed Medical Officer for the Third District of the East Ashford Union. M. H. Hannigan, M.D., R.U.I., M.Ch., appointed Medical Officer for the Seventh and Eighth Districts of the Stow Union. A. Harden, M.Sc., appointed Lecturer in Chemistry at the Pathological Institute of Preventive Medicine, London. F. C. Hitchins, M.R.C.S.Eng., L.R.C.P.Lond., appointed Assistant House Surgeon to the Royal Hants County Hospital, Winchester. R. Hitchings, M.R.C.S., L.R.C.P., appointed Medical Officer of Health of the Headington Union Workhouse. W. A. Hooton, L.R.C.P., M.R.C.S., L.D.S., appointed Honorary Dental Surgeon to the Manchester Royal Infirmary. H. A. Lane, M.R.C.S., L.R.C.P., appointed an Assistant Medical Officer to the Chorlton Union Workhouse. D. Lloyd, L.R.C.P., L.R.C.S.Edin., appointed Medical Officer of Health to the Newcastle Emlyn Urban District. F. W. D. McGachen, L.F.P.S.Glasg., L.S.A., appointed Medical Officer for the Brotton District of the Guisborough Union. E. J. Marmion, B.A., M.B.Dub., appointed Medical Officer of the Melton Union Workhouse. W. P. Montgomery, M.B., B.S.Lond., F.R.C.S.Eng., appointed Resident Surgical Officer to the Manchester Royal Infirmary. P. G. Mould, M.R.C.S.Eng., L.R.C.P.Lond., appointed Third Assistant Medical Officer at the Asylum of the Manchester Royal Infirmary. E. F. M. Neave, M.B., Ch.B.Edin., appointed a Resident Medical Assistant in the Dundee Royal Infirmary. N. Raw, M.D.Durh., appointed Medical Officer to the West Derby Union Infirmary. H. Roscoe, M.R.C.S.Eng., L.R.C.P.Lond., appointed Resident Medical Assistant at the Dyffryn Aled Asylum, near Colwyn Bay. W. Stewart, M.D., appointed Medical Officer for the Bacup No. 2 District of the Harlingden Union. W. H. B. Stoddart, M.R.C.S., L.R.C.P., appointed Pathologist and Assistant Medical Officer to the County Asylum, Prestwich, Manchester. H. Stott, M.R.C.S.Eng., L.R.C.P.Lond., D.P.H.Eng., appointed Medical Officer of Health to the Burgess Hill Urban District. E. W. Sumpter, M.B., B.S.Durh., appointed House Surgeon to the West Norfolk and Lynn Hospital, King's Lynn. J. Sutcliffe, M.R.C.S.Eng., L.R.C.P.Lond., appointed Assistant Medical Officer at Cheadle Asylum. D. Thomas, M.R.C.S.Eng., L.R.C.P.Lond., appointed Medical Officer of Health for Limehouse. J. Torrens, M.D., appointed Medical Officer for the Worfield and Claverley District of the Bridgnorth Union, and Medical Officer for the Trysull District of the Seisdon Union. C. L. H. Tripp, L.R.C.P.Lond., M.R.C.S.Eng., L.S.A., appointed Medical Officer to the Dawlish District of the Newton Abbot Union. O. B. Trumper, M.B., Ch.B., appointed Medical Officer for the Market Rasen and the Tealby Districts of the Caistor Union. J. J. Waddelow, M.R.C.S.Eng., L.R.C.P.Lond., appointed Medical Officer of Health to the Whittlesey Rural District.

